# Supplementary material for: A UMLS-based spell checker for natural language processing in vaccine safety
Source: BMC Med Inform Decis Mak. 2007 Feb 12;7:3. doi: 10.1186/1472-6947-7-3 (PMC1805499; doi:10.1186/1472-6947-7-3)
Supplement: Additional file 13 — RAP application directory. Sets up directory for RAP (RDF application for PHP) source code [file 1472-6947-7-3-S13.gz › rap/doc/phpdoc/__filesource/fsource_utility__utilFindIterator.php.html]

xml version="1.0" encoding="iso-8859-1"?


File Source for FindIterator.php


# Source for file FindIterator.php

Documentation is available at FindIterator.php

```
1. <?php

3. // ----------------------------------------------------------------------------------
4. // Class: FindIterator
5. // ----------------------------------------------------------------------------------


9. /**
10. * Iterator for traversing statements matching a searchpattern.
11. * FindIterators are returned by model->findAsIterator()
12. * Using a find iterator is significantly faster than using model->find() which returns
13. * a new result model.
14. *
15. *
16. * <BR><BR>History:<UL>
17. * <LI>08-05-2004                : First version of this class.</LI>
18. * </UL>
19. *
20. * @version  V0.9.1
21. * @author Tobias Gau� <tobias.gauss@web.de>
22. *
23. * @package utility
24. * @access    public
25. *
26. */
27. class FindIterator extends Object {

29. /**
30. * Reference to the MemModel
31. * @var        object MemModel
32. * @access    private
33. */
34. var $model;


38. /**
39. * Current position
40. * FindIterator does not use the build in PHP array iterator,
41. * so you can use serveral iterators on a single MemModel.
42. * @var        integer
43. * @access    private
44. */
45. var $position;


48. /**
49. * Searchpattern
50. *
51. * @var        Object Subject,Predicate,Object
52. * @access    private
53. */

55. var $subject;
56. var $predicate;
57. var $object;


60. /**
61. * Constructor
62. *
63. * @param    object    MemModel
64. * @param    object  Subject
65. * @param    object  Predicate
66. * @param    object  Object
67. * @access    public
68. */
69. function FindIterator(&$model,$sub,$pred,$obj) {
70. $this->model = &$model;
71. $this->subject=$sub;
72. $this->predicate=$pred;
73. $this->object=$obj;
74. $this->position=-1;
75. }


78. /**
79. * Returns TRUE if there are more matching statements.
80. * @return    boolean
81. * @access    public
82. */
83. function hasNext() {
84. if($this->model->findFirstMatchOff($this->subject,$this->predicate,$this->object,$this->position+1)>-1){
85. return TRUE;
86. }else{
87. return FALSE;
88. }
89. }


92. /**
93. * Returns the next matching statement.
94. * @return    statement or NULL if there is no next matching statement.
95. * @access    public
96. */
97. function next() {
98. $res=$this->model->findFirstMatchOff($this->subject,$this->predicate,$this->object,$this->position+1);
99. if($res>-1){
100. $this->position=$res;
101. return $this->model->triples[$res];
102. }else{
103. return Null;
104. }
105. }


110. /**
111. * Returns the current matching statement.
112. * @return    statement or NULL if there is no current matching statement.
113. * @access    public
114. */
115. function current() {
116. if (($this->position >= -1)&&(isset($this->model->triples[$this->position]))) {
117. return $this->model->triples[$this->position];
118. } else {
119. return NULL;
120. }
121. }

123. }

125. ?>
```

Documentation generated on Thu, 7 Jul 2005 13:41:27 +0200 by phpDocumentor 1.3.0RC3
